# Assessment of Dental Student Satisfaction after Internships in Collaborative Dental Practices in Saxony—A Retrospective Questionnaire Analysis

**DOI:** 10.3390/dj12010014

**Published:** 2024-01-13

**Authors:** Annette Wolf, Mihaela Pricop-Jeckstad, Ute Botzenhart, Tomasz Gredes

**Affiliations:** 1 Department of Prosthetic Dentistry, Faculty of Medicine Carl Gustav Carus, TU Dresden, 01307 Dresden, Germany; annette.wolf@ukdd.de; 2 Department of Applied Mathematics, University Politehnica of Bucharest, 060042 Bucharest, Romania; mihaela.pricop@upb.ro; 3 Department of Orthodontics, Faculty of Medicine Carl Gustav Carus, TU Dresden, 01307 Dresden, Germany; ute.botzenhart@web.de; 4 Department of Orthodontics and Temporomandibular Disorders, University of Medical Sciences, 60-812 Poznań, Poland

**Keywords:** dental education, dental internships, COVID-19, survey

## Abstract

The goal for dental students of a university-based program should be to learn about practice procedures in a dental office as part of their studies in order to gain insight into day-to-day activities, such as organizational management, patient communication, and problem-solving strategies. All dental students from the Faculty of Medicine at the University of Dresden in Germany, who completed a one-week internship in an external dental office in the last year before taking the final exam, were invited to participate in the survey (total *n* = 182 in years 2017–2019 and 2022). After completing the internship, the students were asked to anonymously rate the distinctive competencies they had acquired during their dental studies in terms of clinical and social communication skills. The results of the survey showed a good practicability of the acquired dental knowledge and a general satisfaction of students during their internships. No significant influence of the COVID-19 outbreak and the resulting special regulations in dental practices during the pandemic on student satisfaction was found. Students were more satisfied with their completed internships in smaller cities. Therefore, a stronger inclusion of practices outside the big cities should be considered in the current implementation of the new Dental Licensure Act in Germany.

## 1. Introduction

Professional education of dental students largely includes clinical practice under the supervision of qualified dentists. During their clinical practice, dental students are expected to learn from a variety of sources, including the clinical procedures and the interaction with the dental professionals, the technicians, the patients, and the fellow students. The clinical teachers and dental students often interact on a one-to-one basis, a relationship that has traditionally been one of the most common methods of education [1,2]. However, this process may not adequately prepare the student for professional practice, especially because the clinical learning environment at the university often differs from the daily routine in the dental practice. A current cohort study showed that students in the pre-clinical year prefer the face-to-face modes, especially in Bachelor degree programs and in the transfer of applied knowledge. However, several students also expressed the need for a combination of a face-to-face framework and online teaching to make theoretical learning more effective [3]. In addition to the theoretical part in teaching the dental disciplines, the practical skills that should be used correctly in the treatment of patients are also of considerable importance. Many dental schools around the world organize most of their practical training in their own educational environment. However, there is a recent trend towards improving the practical skills of students in external dental practices, especially in the final year of their education [4,5]. The students in the final phase of their study should better learn how to apply the acquired knowledge and skills in a professional environment, taking into account both clinical and dental treatment and the ability to plan oral health care for patients [5]. In many cases, internships both in cities and in rural areas are organized to cover the full spectrum of dental care throughout the healthcare system and to give an insight into the real everyday life of a dental office with all its facets [4,6].

A multicenter survey conducted at 11 German dental schools indicated deficiencies in the dental education in Germany according to the Dental Licensure Act from 1955 (Zahnärztliche Approbationsordnung—ZAppO). The results of this study showed that such domains as communicative competence, team competence, learning competence, and scholarship were probably not sufficiently integrated into the dental curriculum [7]. A survey of medical students at German universities showed similar results [8]. Corresponding to the new Dental Licensure Act, which has been in effect since 2021/2022, the dental training at German universities should be more practice-oriented. For this purpose, the teaching content and the structure of the training for students is gradually being changed.

The overarching goal of enabling students to practice dentistry independently and under their own responsibility remains in place. In addition, “qualification for further training” is another educational goal. In the future, supplementary knowledge will also be imparted on topics such as the ability to conduct dental discussions with patients, principles of evidence-based evaluation of dental and medical procedures, and the promotion of interdisciplinary thinking; theoretical and clinical knowledge will be introduced together more efficiently in the training. However, these and all other adjustments have no effect on the standard period of study, which remains 10 semesters.

New approaches to medical education have long been considered in many European countries. Over 20 years ago, the Bologna Process initiated the necessary changes in medical and dental education in 46 European countries. However, it is important to recognize that the Bologna Process is more than structural reform. One of the important goals of this reform is the commitment to improving the quality of teaching by moving towards student-centered learning and with a fundamental interest in the learning success of the individual student. It requires curriculum reform based on clearly defined learning outcomes, clarity and quality in teaching, a greater emphasis on student-centered learning, and a greater connection between the university and the practice/market [9].

In order to coordinate a Germany-wide common concept for the additional practical knowledge transfer to students during internships in external dental practices, the representatives of the University of Dresden have devised a model requirement profile with the professional policy institutions in Saxony. Hence, a one-week internship for dental students was started in the final year of their studies, which was set in the lecture-free time between winter and summer semesters. This internship was established by mutual agreement between the medical faculty of the University of Dresden, the Saxon Chamber of Dentists, and more than 80 cooperative dental practices in and around Dresden. After the legal and administrative issues were solved and the ministerial approval was granted, the model of cooperative dental offices integrated into the study program of dentistry was finally implemented in 2016 [10]. A catalogue of learning objectives in the various fields of dentistry was developed for the student activities in this context.

Since there is little knowledge about the self-assessment of the competencies of dental students in Germany [7] and there is a lack of literature on internships in German external dental practices [11], a survey on this topic was designed. The aim of this study was to assess the rating of competencies regarding dental practices in such domains as communication and practical skills, organizational management and problem-solving skills during patient treatment, professional knowledge, and openness to criticism of Saxon dental students.

The hypotheses of this study were as follows:

(i) the levels of the competencies self-assessed by the students prior to final exams in the mentioned domains regarding the future professional life would be rated as satisfactory;
(ii) no significant deviations were expected in the survey between male and female students;
(iii) no significant differences in these ratings were expected after completing an urban or rural internship.

This survey started before the outbreak of the pandemic. This allowed an additional comparison of the students’ answers before and after the new hygiene regulations and few restrictions in dental practices and at universities with regard to COVID-19.

## 2. Materials and Methods

### 2.1. Study Design and Participants

Data for this study were collected over four years (2017, 2018, 2019, 2022). Before starting, the study was approved by the Ethics Committee (reference number BO-EK-100022023) in accordance with the guidelines of the Declaration of Helsinki. All dental students from the Faculty of Medicine at the University of Dresden in Germany, who completed a one-week internship in an external dental office in the last year before taking the final exam, were invited to voluntarily participate in the survey (*n* = 52 in 2017; *n* = 40 in 2018; *n* = 52 in 2019; *n* = 38 in 2022; total *n* = 182). The choice of dental office was left to the students who were enrolled in the practice program at the University of Dresden. After completing the internship, final-year students were asked to anonymously rate their satisfaction with clinical and social skills in the dental offices. Participation was both anonymous and voluntary, hence ensuring the anonymity of all data.

### 2.2. Questionnaire

**Figure 1:**
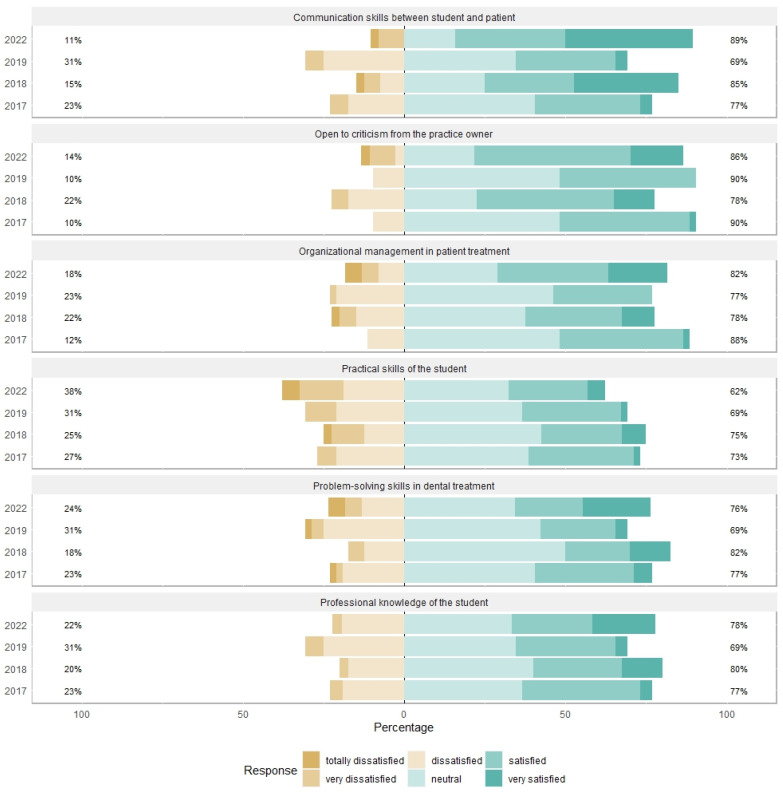
Likert plot of the grading scores by variable and by year.

The questionnaire in German was developed specifically for this study by the dental staff of the Department of Prosthetics at the Medical Faculty of the Technical University of Dresden. It included information regarding gender, year of study, location of dental practice, and six other questions. Six questions were formulated to be as simple as possible. The first three questions of the survey related to students’ new experiences in a new work environment. These questions were asked about communication skills between student and patient, organizational management in patient treatment, and students’ openness to criticism from the practice owner. The other three questions asked students about their knowledge of both theoretical and practical dentistry and their ability to solve problems during patient treatment (Figure 1).

The satisfaction survey elicited responses on a six-point Likert-type scale for the six questions. At the end of the questionnaire, students were allowed to provide qualitative feedback on their experience.

### 2.3. Statistical Analysis

The probability distributions of the grading scores available for the five variables corresponding to our questionnaire items for the years 2017, 2018, 2019, and 2022 were displayed in a Likert plot separately for each question. The chosen Likert scale had a starting value of 1 (= totally dissatisfied) and reached a maximum of 6 (= very satisfied).

Afterwards, these scores were treated as numerical values, and their yearly five-number summary (the minimum, the maximum, the median, and the 25% and 75% quantiles), as well as the yearly mean, standard deviation, standard error, and sample size, were also computed. Various linear regression models were used to estimate the relationship between our variables and the covariates such as year, town size, and sex, and their interactions. Town size was a binary variable with a category “small” for localities with less than 40,000 residents and a category “large” otherwise. Moreover, another regression analysis was also applied to detect a “corona effect” by replacing the year with a binary variable with categories “before” and “after” 2020. For both families of regression models, we considered the Akaike information criterion (AIC) for the model selection. The results of the regression analysis are displayed as the effect sizes for the chosen models, together with their confidence intervals and their statistical interpretation. The statistical analysis was performed using R Statistical Software (v4.3.0; R Core Team 2022).

## 3. Results

The probability distributions of the Likert scores are positively skewed, and differ between the years included in the study (see Figure 1). In general, both the percentages and the location parameters indicate at least a neutral satisfaction with the students’ skills and professional situation (see Table 1 and Figure 1). The practical skills in 2022 are, as expected, less graded compared to the other years (a mean of 3.75 versus 3.94, 4, and 4.04 in previous years) but there was no recognizable trend in the evaluation of the study items (see Table 1).

The linear regression models including the year, town size, and gender, and their interactions, revealed a paradigm of at most two independent variables associated with each independent variable after applying AIC for model selection, as displayed in Table 2. This might be due to the small sample size for each year. Based on the statistical analysis, the students’ communication quality was related to the year of the internship and the size of the city. Openness to criticism as well as practical and theoretical knowledge correlated with gender and city size. In addition, organizational skills and problem-solving quality were associated with city size. It seems that students were, in general, more satisfied with the internships in the small towns. Moreover, the town size was a statistically significant effect for all questions graded in the study. Other statistically significant effects were the year for the communication skills, with an improvement in 2022 over 2019, and the gender for the openness to criticism, with men grading this quality more highly compared to women.

We also looked for a “post-Corona” effect and considered regression models including a Corona binary variable instead of the year, town size, and gender, and their interactions, as shown in Table 3. The paradigm changed only slightly and at most two independent variables associated with each dependent variable were kept after applying AIC for model selection. The communication quality and the organizational skills were associated with the post-Corona effect and the town size; the openness to criticism and the practical and theoretical knowledge with gender and the town size; and the problem-solving quality with the town size. The students working in small localities were, in general, more satisfied with the items that were graded, and the town size had a statistically significant effect on all questions graded in the study, as seen in the previous statistical analysis. The Corona effect was statistically significant for the communication skills, with an improvement in 2022 over the whole period 2017–2019, and gender was statistically significant for the openness to criticism, as shown before.

## 4. Discussion

Dental education enables prospective dentists to acquire the knowledge and skills necessary to provide people with the best possible dental treatment. Care must be taken to ensure that students are provided with a satisfactory work environment throughout their education. The results of many studies indicated that high psychological stress and lack of hands-on training could be a growing problem among dental students [12,13]. Many medical and dental students at German universities had higher stress levels because they were particularly concerned about the progress or quality of their education [14,15]. The learning environment and real dentistry also seem to play an important role for students [16]. Therefore, we wanted to investigate how comfortable and confident students feel during a short internship near the end of their dental education.

The first hypothesis of this study, that the competencies assessed by the students in the last year of study would be rated as satisfactory for their future professional life, was confirmed. The student’s skills in the areas of communication with the patients and practical skills, organizational management, and problem-solving skills in patient treatment, professional knowledge, and openness to criticism in Saxon dental practices were predominantly in the neutral or positive range of the answers given. Although these results were mostly considered satisfactory, consistently positive evaluations in the examined domains were not to be expected based on a previous multicenter study in Germany, which showed certain deficits in the training of dental students. Dental students in their last clinical year of many German dental schools took part in those surveys to determinate their communicative competence, team and learning competences, and scholarship using the Freiburg Questionnaire to Assess Competencies in Medicine. The results of all participating dental schools revealed several deficiencies in all domains of competencies [7].

The assessments of the presumed level of necessary competencies that students consider for their job were significantly lower in comparison to the level of competencies in the final year of study [7]. We were able to show a different view of the students regarding university preparation for their working life in contradiction to the previous assessments. However, it should not be forgotten that our questions were closely related to the one-week internship in the dental practice and not generally related to the dental education at the University of Dresden. Our results largely corresponded with the results of a cooperation between another German university and general dental teaching practices, where both students and dental practitioners demonstrated a high level of satisfaction concerning the shadowing [11]. The seventh-semester students felt their communication and social competencies improved when dealing with patients and the practice team. The greatest progress was perceived to occur in the areas of accounting and practice organization, as well as dentist’s discussion techniques [11]. Although the quality of learning and the methods of assessment appear to be quite variable, a review of the communication skills in dental education indicated that most dental students are open to learning new communication skills. The prospective young dentists are aware that these skills are an essential element of the patient–dentist relationship, mainly because the patients exhibit many different communication styles, often related to their trusting or anxious behavior [17]. Studies have shown that students of dentistry and medicine can expand their skills in external practices, where experienced colleagues can convey information and requirements for new areas in a simple and understandable way [18,19,20]. Although the mediators of theoretical and practical knowledge in the present study were mostly dentists without training or teaching experience, the results and the high level of student’s satisfaction showed that these short internships were nevertheless successful. In a recent review, dental students found supervision and communication most effective when the hierarchy between them and their supervisors was flattened [16].

The students often regard the dentists as motivating role models and acknowledge a growth in knowledge and an improvement in their behavior in dealing with patients and the practice team [11]. Therefore, they can deal well with the constructive criticism of experienced colleagues, which was also proven in our study. Although we did not expect any significant differences in the answers to our questionnaire between the female and male students at the beginning of the study, it was found that the male students were better able to deal with criticism in the practices than their female fellow students. Results of a study of emotional intelligence and perceived stress in dental undergraduates showed that females scored higher regarding emotions and social skills, but did not show significant differences for optimism/mood regulation [21,22,23]. Many studies, including multi-country studies, also showed differences in the way female and male dental students deal with stress. Female students often exhibited significantly more anxiety and stress than male participants in student education courses, which is also reflected later in professional lives of working dentists [12,24]. Gender-specific differences were found both in the coping dimensions and in the individual coping strategies used [17]. However, until now, it was not possible to clearly determine whether female dental students are more sensitive to certain aspects of the educational process or exhibit different patterns of educational morbidity, or males are less expressive of their concerns [25,26,27].

Since we evaluated the survey on student internships in different dental practices in Saxony, the question arose whether students in urban and rural practices were comparably satisfied. This is an interesting aspect, considering that fewer and fewer dentists in Saxony are deciding to establish a practice or to take over an existing one in rural areas. A strong disparity in the distribution of dentists between rural and urban areas seems to also be a problem in many countries [28]. Some international studies suggest that students should have more chances to witness the rural dentists’ offices and experience rural life in order to attract graduates to work in these areas after graduation. The experiences in small rural dental offices could correct misconceptions and prejudices and inspire students to work outside of big cities [29]. The dental school curricula that include rural rotations could increase students’ sensitivity to the issues regarding the patients and increase students’ likelihood of choosing a dental practice in the countryside [30]. The positive experiences of dental students during short-term practice in the countryside or outside the big cities have often been reported in the literature [28,29,30]. These observations were also confirmed in our survey, where the respondents’ satisfaction with the one-week internship in dental offices in smaller cities or rural areas was significantly higher than that in big cities.

It should be remembered that dental students’ interests during their undergraduate studies often change in their postgraduate plans. Some studies have shown that almost half of the students changed their focus for professional life while progressing towards graduation [31]. Hence, a wide spectrum in the teaching of dental students, including internships with patients in the dental practices outside the university facilities in large and small towns or villages, had an influence on the choice of the place for the further professional life or the settlement after their graduation [32].

Many studies have also found that both dental and medical students benefit from and understand the importance of short internships during their studies. One of these studies reveals that the medical students of the last three years not only consider their rural internship to be useful shortly before the end of their studies, but also prefer such internships at the beginning of their studies, understanding the advantages and importance of such placements [33]. Furthermore, the introduction of mandatory clinical rotation in rural areas during dentistry studies seemed to be effective to counteract the shortage and maldistribution of dentists in rural areas [34].

Some surveys at European universities have proven that the dental students possessed excellent theoretical knowledge as well as basic clinical skills; however, they were still lacking in the experience of complex treatments, which could lead to a reduced willingness for independent practice [35]. Professional practices during their dental education also offered them options for their professional future that they have not yet decided on [36]. European dentistry students mainly aim to become self-employed and work in their own practice. It has been found that dentists working in small groups reported the highest overall satisfaction compared to colleagues working in large teams. On the other hand, dentists who work in large groups reported being more satisfied with their income and services than dentists in individual practices. According to the most recently published meta-analyses on dentist job satisfaction, which were based on global data, dentists were moderately to highly satisfied with their work, with specialists being more satisfied than general dentists. The most satisfying factors in relation to the work environment were patient relationships, respect, delivery of care, staff, professional relationships, and professional environment [37]. All of these factors could be faced by the students participating in our study during their internships, and the evaluation of the survey showed that the internships completed in the smaller cities were rated significantly better. It is therefore important to encourage students to also gain work experience outside of the big cities during their studies. Thus, the size of cities, practices, and staff could have a decisive influence on later professional decisions. 

With the outbreak of the COVID-19 pandemic, the daily routine in dental practices changed and the students’ internships also had to be adapted to the new hygiene concept requirements. The question arose as to whether student satisfaction with placements during this period was also comparable to that prior to the outbreak of the pandemic. The students’ answers to our questionnaire from 2021 therefore were analyzed considering a new reality of patient care under the special hygiene and protective measures. The first study to assess the mental effect of the COVID-19 pandemic on German dental students in 2021 showed an overall normal or mild psychological impact of the pandemic on anxiety, stress, depression, intrusion, hyperarousal, and avoidance [38]. A recent Norwegian study showed that the strong stressors related to the new study situation during the COVID-19 pandemic also affected female students more often. According to our survey both before and during the pandemic, a difference in the handling of criticism between male and female dental students could also be proven. Thus, the male students were better able to deal with criticism than their female fellow students throughout the study. The students’ clinical and theoretical learning outcomes were often rated worse than before the pandemic [39,40], which was also reflected as a trend in the results of our study. Practical skills were rated lower in 2022 than in previous years, presumably due to the fact that students spent less time treating patients as a result of numerous safety and hygiene measures. The communication skills during students’ one-week internship were found to be better by the participants after the outbreak of the pandemic. This may indicate an effort by both the higher education institutions and the students to ensure direct contact with patients, which could not be replaced by additional phantom exercises.

Our survey has certain limitations in terms of accurately assessing the internship. Compared to a thematically similar German study [7,11], the questionnaire in our study was formulated for students in a short and simplified form. It was not subdivided into sections for the student’s self-assessment before and after the period in the practice, or for an external assessment by the practicing dentist, as has been done, for example, in other studies [11]. Mutual assessment would certainly be an interesting aspect to put the students’ statements in perspective. In addition to the questions asked, which had to be answered on the satisfaction scale, the students were asked to write a freely structured report in which they could describe and comment on the internship process, as well as the experiences and insights they gained. Since the survey was voluntary, and despite the request to fill out the forms completely, participants often omitted the additional fields. In the answers provided, the frequent negative feedback was that the internship period was far too short and the students were not given enough independent work. The freely structured reports illustrated a largely positive viewpoint of the students, who often mentioned a good opportunity for gaining insight into the daily life of a practice, interesting patient cases, the possibility of treating patients independently, and friendly and courteous staff in dental offices.

Another shortcoming of our survey was the lack of information about the size of the dental practices, their equipment, the number of patients treated in the practice week, or what detailed types of treatments were performed on patients by the students. This precise information would certainly be helpful in data analysis regarding differences be-tween practices in large and small towns. Furthermore, regarding the small town group, no other distinction was made between practices in small towns and rural practices.

## 5. Conclusions

Despite the simplification of our survey, a clear conclusion could be drawn that city size has an important influence on all the issues assessed in the study. Students were generally more satisfied with internships in small towns. City size had a significant impact on all questions assessed in the study, as seen in the previous statistical analysis. Differences in handling criticism were observed, with male students being more open to criticism than female students. Given the current trend of young dentists leaving small towns and preferring to settle in large cities, this information should encourage students not only to complete their internship in small towns or villages, but also to work there full-time later on. Although the impact of the unpredictable COVID-19 pandemic on internships was not initially considered in this study, the COVID-19 effect proved to be significant for students’ communication skills, which improved after the pandemic subsided. 

It is expected that the practical part of the training, which has been expanded as part of the new Dental Licensure Act, will further increase the satisfaction among students and prepare them even better for their professional lives.

## Figures and Tables

**Table 1:** Summary statistics by variable and year.

Variable	Median	Mean	SD	SE	Min	Max	25%	75%	Median	Mean	SD	SE	Min	Max	25%	75%
**Year**	**2017**								**2018**							
**Organizational skills**	4	4.30	0.70	0.09	3	6	4	5	4	4.17	0.70	0.17	1	6	4	5
**Practical knowledge**	4	4.03	0.92	0.12	2	6	3	5	4	4	0.92	0.18	1	6	3.75	5
**Professional knowledge**	4	4.17	0.92	1.12	2	6	4	5	4	4.30	0.99	0.15	2	6	4	5
**Communication skills**	4	4.11	0.94	0.13	2	6	4	5	5	4.67	1.28	0.20	1	6	4	5
**Openness to critics**	4	4.34	0.68	0.09	3	6	4	5	5	4.40	1.08	0.17	2	6	4	5
**Problem-solving skills**	4	4.13	0.99	0.13	1	6	4	5	4	4.22	0.99	0.15	2	6	4	5
**Year**	**2019**								**2022**							
**Organizational skills**	4	4.05	0.77	0.10	2	5	4	5	5	4.36	1.32	0.21	1	6	4	5
**Practical knowledge**	4	3.94	0.99	0.13	2	6	3	5	4	3.75	1.27	1.20	1	6	3	5
**Professional knowledge**	4	4.01	0.98	0.13	2	6	3	5	4	4.34	1.11	1.18	2	6	4	5
**Communication skills**	4	4.01	0.98	0.13	2	6	3	5	5	4.89	1.31	1.21	1	6	4.25	6
**Openness to critics**	4	4.32	0.64	0.08	3	5	4	5	5	4.53	1.19	1.19	1	6	4	5
**Problem-solving skills**	4	3.92	0.98	0.13	1	6	3	5	4	4.25	1.36	0.22	1	6	4	5

**Table 2:** Statistical effects in the linear regression models by variable (flagged values are statistically significant effects). Intercept^ represents the mean of the year 2017 in the small towns for communication skills, organizational skills, and problem-solving skills, and is the mean of the year 2017 in the small towns for men for the rest of the dependent variables. (Significance codes for the *p*-values: 0 ‘***’ 0.001 ‘**’ 0.01 ‘*’ 0.05 ‘ ’ 0.1 ‘ ’ 1).

	Communication Skills	Openness to Critics	Organizational Skills	Practical Knowledge	Problem-Solving Skills	Professional Knowledge
**Intercept^**	4.48 *** [4.11, 4.86]	4.42 *** [4.11, 4.72]	4.65 *** [4.39, 4.91]	4.66 *** [4.31, 5.01]	4.57 *** [4.28, 4.86]	4.35 *** [4.01, 4.69]
**Town_size_1**	−0.60 ** [−0.96, −0.24]	−0.38 * [−0.67, −0.09]	−0.59 *** [−0.90, −0.29]	−0.75 *** [−1.09, −0.42]	−0.63 *** [−0.97, −0.29]	−0.48 *** [−0.80, −0.16]
**Year_2018**	0.64 *** [0.19, 1.09]	−	−	−	−	−
**Year_2019**	−0.02 [−0.44, 0.41]	−	−	−	−	−
**Year_2022**	0.88 *** [0.42, 1.35]	−	−	−	−	−
**Gender_w**	−	0.34 [0.04, 0.63]	−	−0.24 [−0.58, 0.10]	−	−0.25 [−0.08, 0.58]
**N**	128	128	128	128	128	128
**R^2^**	0.15	0.05	0.07	0.12	0.07	0.12

**Table 3:** Statistical effects in the linear regression with Corona effect by variable (flagged values are statistically significant effects) Intercept^ represents the mean in the small towns before the COVID-19 pandemic for communication skills, organizational skills, and problem-solving skills, and is the mean for men in the small towns for the rest of the dependent variables. (Significance codes for the *p*-values: 0 ‘***’ 0.001 ‘**’ 0.01 ‘*’ 0.05 ‘ ’ 0.1 ‘ ’ 1).

	Communication Skills	Openness to Critics	Organizational Skills	Practical Knowledge	Problem-Solving Skills	Professional Knowledge
**Intercept^**	4.48 *** [4.11, 4.86]	4.42 *** [4.11, 4.72]	4.65 *** [4.39, 4.91]	4.66 *** [4.31, 5.01]	4.57 *** [4.28, 4.86]	4.35 *** [4.01, 4.69]
**Town_size_1**	−0.57 ** [−0.93, −0.20]	−0.38 * [−0.67, −0.09]	−0.61 *** [−0.92, −0.30]	−0.75 *** [−1.09, −0.42]	−0.63 *** [−0.97, −0.29]	−0.48 *** [−0.80, −0.16]
**Coeffect**	0.71 *** [0.31, 1.11]	−	0.24 [−0.10, 0.58]	−	−	−
**Gender_w**	−	0.34 * [0.04, 0.63]	−	−0.24 [−0.58, 0.10]	−	−0.25 [−0.08, 0.58]
**N**	128	128	128	128	128	128
**R^2^**	0.10	0.05	0.08	0.12	0.07	0.05

## Data Availability

The original contributions presented in the study are included in the article, further inquiries can be directed to the corresponding author.
